# Distortion of an LTCC Bilayer during Constrained Sintering: Comparison between Ombroscopic Imaging and Modeling

**DOI:** 10.3390/ma15186405

**Published:** 2022-09-15

**Authors:** Lucie Chrétien, Adrien Heux, Guy Antou, Nicolas Pradeilles, Nicolas Delhote, Alexandre Maître

**Affiliations:** 1 University Limoges, CNRS, IRCER, UMR 7315, F-87000 Limoges, France; 2 University Limoges, CNRS, XLIM, UMR 7252, F-87000 Limoges, France

**Keywords:** glass ceramics, constrained sintering, distortion, ombroscopy, finite element method

## Abstract

A complete methodology combining experiments and modeling has been developed to investigate the constrained sintering of low-temperature cofired ceramic (LTCC) systems. The thermomechanical and sintering behavior laws, previously identified for the selected commercial LTCC material, were implemented in a finite element model. The reliability and validity range of the built model has been investigated thanks to the development of a specific distortion experience. The distortion generated during the constrained sintering of a porous LTCC layer deposited on a dense one has been monitored in situ by ombroscopy. The measured camber evolution was compared with numerical results. The camber phenomena predicted numerically and observed experimentally are very similar, characterized by the onset of distortion around 918 K and a similar evolution during heating. However, at high temperatures (around 1100 K), the simulated camber slightly differs from the experimental one. It seems to be related to the damage to the dense LTCC layer by microcracking.

## 1. Introduction

Elaboration of multilayer components combining ceramic and metal materials for microchip packaging applications is an important strategy of research development [1]. Low-temperature cofired ceramics (LTCCs) are materials exhibiting low dielectric loss as well as good adherence with thin and highly conductive metallic films (silver, gold) [2,3]. These performances permit the creation of very compact packages for passive microwave and millimeter wave components.

In the fabrication process of LTCC devices, the thermomechanical compatibility between metallic and ceramic layers is a key point to be controlled during co-sintering [4,5]. The layers being of different nature, their densification kinetics, as well as their thermal expansion coefficients can differ, thus inducing the generation of stresses at the interfaces between materials. For example, considering a bilayer composed of material 1 and material 2, if the densification rate of 1 is greater than that of 2, 1 will be in tension while 2 will be in compression [4,5]. The stresses generated tend to slow down or accelerate the individual densification of each layer, leading to damage (cracking, delamination) or anisotropic shrinkage, characterized by the distortion of one or more layers [6,7]. To minimize these problems related to co-sintering, the first way of research consists of developing materials with similar deformation behavior. Thus, in the LTCCs family, new chemical compositions are developed to favor the thermomechanical compatibility between metallic and ceramic layers while improving dielectric performance [8,9]. In parallel, the behavior of LTCC films during constrained sintering is investigated to understand their densification kinetics and microstructural anisotropy potentially induced [10,11]. The complementary way to improve and control the critical co-sintering stage involves modeling the thermomechanical behavior of the interacting layers. Modeling can participate in the design of LTCC-based systems (e.g., dimensions and geometric shapes of different layers) in order to manufacture components with reproducible performances and a predictable shrinkage.

Analytical models have been developed to estimate the distortion generated during co-sintering. During the expansion phase at low temperatures, in the particular case of a bilayer with a planar interface, a simple thermoelastic model makes it possible to calculate the curvature generated by knowing Young’s moduli and the expansion coefficients of the materials involved. Guillon [12] applied this thermoelastic model to predict the camber of a bilayer composed of an alumina layer deposited on dense platinum foils. A good correlation with experimentation was noticed at low temperatures, i.e., before the densification process. At higher temperatures during the densification stage, Cai et al. [13] estimated the curvature generated during the co-sintering of an alumina-zirconia bilayer based on the densification rates differential between the two layers. Similarly, Kanters et al. [14] proposed an analytical model to simulate the curvature during co-sintering of laminates consisting of two different nanocrystalline zirconia materials (undoped and 3Y-TZP doped).

The analytical approaches mentioned above are limited to simple geometry, i.e., to a bilayer component with a planar interface. They are unsuitable for understanding and predicting damage and distortion phenomena generated during constrained sintering or even co-sintering of objects with complex three-dimensional shapes. Mastering this co-sintering step—a key point in additive technologies—requires the development of numerical models integrating representative thermomechanical constitutive laws [15,16,17]. The main objective is to minimize the costly trial-and-error step. Huang et al. [15] applied the finite element method (FEM) to predict the shape evolution of multi-layered films during constrained sintering. Considering a series of case studies in constrained sintering, they compared numerical results given by different models, i.e., an empirical numerical method vs. models based on sintering constitutive laws proposed by Du and Cocks [18] or Olevsky [19]. No comparison with experimental data in constrained sintering was performed. Ou et al. [16] developed a finite element model to simulate the co-sintering of bi-material components made of 316L stainless steel and copper. A linear isotropic viscous behavior (Newtonian type) was used, and the viscous parameters were identified by bending tests. Numerical results were validated using measured shrinkage data only. More recently, Rasp et al. [17] used FEM and the discrete element method (DEM) to simulate shape deformation and cracking of cylindrical cavities within alumina bodies. Post-mortem observations of cavities and surrounding cracks were carried out and compared to the model prediction.

The validation step of numerical models simulating constrained sintering is a key point that requires the development of appropriate experimental tools. A few studies have used ombroscopic imaging to investigate the phenomenon of distortion during constrained sintering [7,12,20]. This method provides contactless access to the measurement of shape changes and dimensional variations of sintered bodies (in both directions of the viewing plane). As an in situ monitoring of shape deformation, the ombroscopic method allows comparison between models and experiments throughout imposed sintering thermal cycle. It is hence a well-adapted way to check the reliability of numerical models.

In a previous work [21], we carefully characterized the thermoelastic, viscoplastic, and sintering properties of a commercial LTCC material (ref. ESL 41111-G). The present work aims to develop a complete methodology combining experiments and simulation in order to investigate the behavior of LTCC layers during constrained sintering. First, a numerical model integrating the previously-characterized thermomechanical data is proposed. The robustness of the developed numerical model is validated through comparison with a specific distortion experience. The constrained sintering of a green LTCC layer deposited on a dense one is studied. An original method of in situ monitoring of the camber of the bilayer is developed by ombroscopic imaging. The reliability of the built numerical model is discussed by comparing the values recorded experimentally with those calculated. The validity range of the model is defined and discussed.

## 2. Materials and Methods

### 2.1. Processing of the LTCC Bilayer

In this study, commercial LTCC tapes of 100–130 × 10^−6^ m thick were used (ref. ESL 41111-G, ESL Electroscience, King of Prussia, PA, USA). In a previous work [21], its microstructure was carefully characterized using different analysis methods. This LTCC consists of micrometric grains of rhombohedral quartz embedded in an amorphous silicon-based phase. A green density of 2020 ± 10 kg·m^−3^ and a bulk density of 2240 ± 10 kg·m^−3^ (after sintering) were measured by a helium pycnometer.

**Figure 1:**
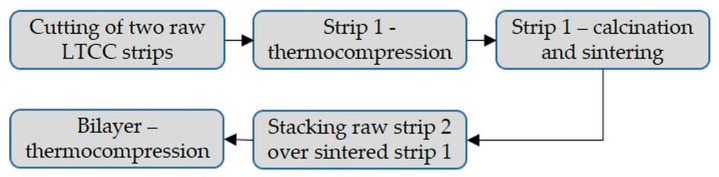
Synoptic summarizing the elaboration protocol of the LTCC bilayer.

Figure 1 presents the protocol used to manufacture the cohesive bilayer material composed of a porous LTCC layer over a dense one. First, two strips of LTCC were cut to obtain an area of 0.2 × 0.2 m^2^. The first strip was prepared by thermocompression at 343 K under uniaxial stress of 21 × 10^6^ Pa. Then, the strip is calcined in air at 723 K for 3600 s. Finally, this layer is sintered at 1123 K for 600 s with a heating rate of 0.083 K/s (i.e., 5 K/min). These two heat treatments are applied in a Ceradel furnace (C9DL, Ceradel, Panazol, France). Once sintered, the thickness of the strip, measured using an interferometric microscope (ZOOMSurf 3D, Fogale Nanotech, Nîmes, France), is equal to 90 ± 1 × 10^−6^ m and appears to be homogeneous on the surface.

Then, the second strip of raw LTCC was deposited on the previously sintered one. The same thermocompression cycle involving an applied load of 21 × 10^6^ Pa at 343 K was carried out in order to obtain a cohesive bilayer.

**Figure 2:**
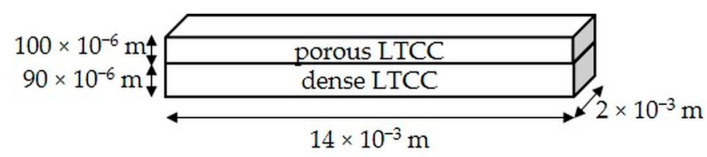
Geometry of the LTCC bilayer.

After this thermomechanical treatment, the total thickness of the bilayer measured by the same interferometric microscope was equal to 190 ± 1 × 10^−6^ m. Finally, rectangular bars of 0.014 m long and 0.002 m wide were cut using a diamond wire saw (STX 202A Diamond Wire Saw, MTI Corporation, Richmond, CA, USA). Figure 2 presents the final geometry of the elaborated LTCC bilayer.

### 2.2. Camber Measurement by Ombroscopy

In order to perform the camber measurement, a heat treatment is applied to the manufactured bilayer in a tubular furnace (Carbolite HVT 15/75/450, Carbolite, UK). Moreover, during this treatment, the bilayer is placed on an alumina support. The reference thermal cycle suggested by the supplier of the LTCC material is applied, i.e., calcination in air at 723 K for 3600 s followed by sintering at 1123 K for 600 s (with a heating rate of 0.083 K/s). During this heat treatment, a distortion is generated by sintering the green LTCC constrained by the dense layer. The camber of the sample depends on the parameters of the heat treatment (temperature and time). So, during the heat treatment, two steps are observed:

1. At low temperatures, dilatation of the sample is uniform, and no camber occurs;
2. At high temperatures (above 973 K), porous LTCC begins to densify, generating compressive stresses within the dense LTCC. Due to the low rigidity of the bilayer linked to its low thickness, a camber appears, allowing the relaxation of stresses generated by the densification of the porous LTCC.

**Figure 3:**
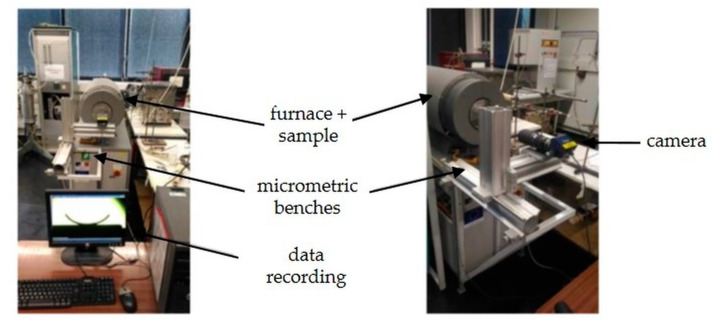
Developed device of ombroscopy.

An ombroscopy system is developed to monitor in situ the induced camber (Figure 3). The ombroscopy system is composed of micrometric benches allowing the precise positioning of the camera (ProgRes C7, JENOPTIK, Jena, Germany, 7 megapixels) in the three directions of space. This one is associated with a lens (AF MCRO-NIKKOR 200/F4 D IF-ED, Nikon, Nikon Europe), which results in a resolution of the acquisition system around 2.7 × 10^−6^ m per pixel. The data are recorded using a computer connected to the furnace and to the camera, which allows one to take pictures for a time step defined by the user or for a given temperature variation. Here, one image is taken per minute or every 5 K.

Obtained images are then analyzed using ImageJ software (ImageJ, version 1.51, National Institutes of Health, Bethesda, Bethesda, MD, USA) to determine the radius of curvature of the sample as follows (Equation (1)):

(1) $$\left|R\right|=\frac{r^{2}+h^{2}}{2h}$$

where *r* and *h* are, respectively, the horizontal and vertical displacements of the endpoint of the bilayer measured using the ImageJ software.

The camber is thus obtained by this Equation (2):

(2) $$C=\frac{1}{\left|R\right|}$$

## 3. Camber Modeling

Thermomechanical and sintering constitutive laws of the LTCC material were determined in our previous study [21]. They are implemented in a numerical model using Comsol Multiphysics software (version 5.6, Grenoble, France) in order to simulate the constrained sintering behavior of the bilayer material.

The thermomechanical model used is based on the heat equation (Equation (3)) and the momentum conservation equation (Equation (4)), in which gravitational and inertia effects are neglected:

(3) $$\rho_{\nu }c_{p}\frac{\partial T}{\partial t}=k{𝛻}^{2}T$$

(4) 𝛻σ=0

with *ρ*_*ν*_ the density, *c*_*p*_ the heat capacity, *k* the thermal conductivity, *T* the temperature, *t* the time, and σ the stress tensor.

During the thermal treatment, the overall deformation of the LTCC material can be decomposed into two categories: (i) reversible thermo-elastic deformation and (ii) irreversible deformation associated with the phenomena of sintering or/and creep. The irreversible strain rate of the LTCC material (${\dot{\varepsilon }}_{i}$) is therefore expressed as follows (Equation (5)):

(5) $${\dot{\varepsilon }}_{i}={\dot{\varepsilon }}^{fr}+{\dot{\varepsilon }}^{vp}$$

with ${\dot{\varepsilon }}^{fr}$, the strain rate related to the sintering process and ${\dot{\varepsilon }}^{vp}$, the viscoplastic strain rate (associated with creep).

During the densification process, the closure of pores leads to the variation of apparent volume and, therefore, relative density (*ρ*). The mass conservation equation is described as follows (Equation (6)):

(6) $$-\frac{1}{\rho }\frac{\partial \rho }{\partial t}=\dot{e}$$

where $\dot{e}$ is the first invariant of the strain rate tensor.

The free sintering behavior of the LTCC material was previously analyzed and modeled using the MSC method [21]. It leads to the following expression giving the evolution of the relative density as a function of time and applied temperature [22] (Equation (7)):

(7) $$\rho =\rho_{0+}\frac{\left(1-\rho_{0}\right)}{1+exp\;\left(-\frac{\left(\log\;\left(\Phi \right)-b\right)}{c}\right)}$$

where *b* and *c* are constants to be fitted, *ρ*_0_ is the initial relative density. The function *Φ(t,T(t))* is defined as follows (Equation (8)):

(8) $$\Phi \left(t,T\left(t\right)\right)=\overset{t}{\underset{0}{\int }}\frac{1}{T}exp\left(-\frac{Q_{fs}}{RT}\right)dt$$

with *Q*_*fs*_ the apparent activation energy of free sintering and *R* the gas constant.

The creep behavior of the LTCC material has been previously characterized [21] using a linear isotropic viscous behavior (Newtonian type) [23,24]. So, the viscoplastic strain rate can be expressed as follows (Equation (9)):

(9) $${\dot{\varepsilon }}_{i}^{vp}=\frac{1}{\eta }\;\left(\sigma_{i-}\;\nu_{p\;}\left(\sigma_{j+}\sigma_{k}\right)\right)$$

with *η* the uniaxial viscosity, *ν*_*p*_ the viscous Poisson’s ratio, σ_i_ the normal stress along the direction *i* and *i,j,k* = 1,2,3.

These two viscoplastic constants (*η, ν_p_*) were previously identified [21]. The viscous Poisson’s ratio was determined by the methodology suggested by Mohanram et al. [24]. A low and stable value was obtained, with an average value around 0.14 ± 0.01 for 0.6 < *ρ* < 0.9. The uniaxial viscosity was determined using cyclic loading dilatometry, yielding values from 0.1 to 2 × 10^9^ Pa·s. The evolution of uniaxial viscosity as a function of temperature and relative density is described by Equation (10):

(10) $$\eta =\frac{\eta_{0}\;exp^{\left(\frac{Q_{\nu p}}{RT}\right)}exp^{\left(\beta \rho \right)}}{T}$$

with *η*_0_ a pre-exponential factor, *Q*_*vp*_ the apparent activation energy of viscoplastic behavior and *β* a constant.

Table 1 lists the main thermomechanical characteristics of the LTCC material previously determined [21] and considered here for numerical simulation.

The evolutions of the elastic constants (i.e., Young’s modulus and Poisson’s ratio) as a function of temperature and relative density have been implemented in the numerical model based on the previously measured data [21]. The thermal expansion coefficient of both layers (dense and porous) is considered identical at approximately 3 × 10^−6^ K^−1^ [11].

Thanks to the symmetry of the bilayer sample, its geometry in simulation is reduced to only half of its length, i.e., a length of 7 × 10^−3^ m, a width of 2 × 10^−3^ m, and an initial total thickness of 190 × 10^−6^ m. This geometry is finely meshed (128,000 elements of free tetrahedron type) because of the low thickness of the layers with respect to their lengths and widths. The following boundary conditions are imposed at the level of the plane of symmetry:

1. A zero displacement is imposed in the out-of-plane direction (with respect to the bilayer joining plane) on the edge common to both LTCC layers;
2. A zero-displacement imposed in the in-plane direction (width axis) on one of the two extreme points of the former edge.

## 4. Results and Discussion

### 4.1. Free Sintering

**Figure 4:**
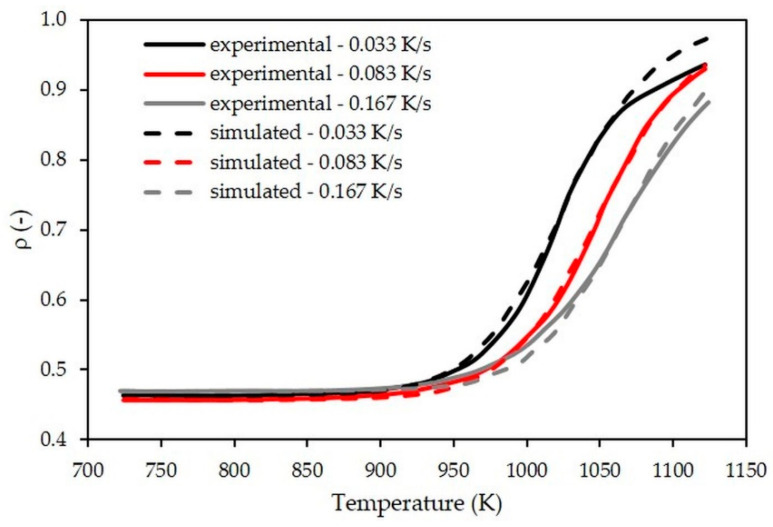
Comparison between simulated and measured densification curves during free sintering for different heating rates applied up to 1123 K.

Using the constitutive equation (Equation (7)) defined for free sintering using the MSC method, the densification curves for various heating rates from 0.033 to 0.167 K/s up to 1123 K have been numerically simulated and compared with the experimental curves (Figure 4). A good correlation at the early and intermediate stages of sintering is observed for the three heating rates applied. A greater difference is noticed at the final stage of sintering and, more particularly, for the test with the ramp of 0.033 K/s. However, the difference in terms of final relative density remains acceptable (around 3%). This is explained by the fact that the parameters of the MSC law have been optimized for the reference thermal cycle, during which the final stage of sintering is not yet reached, unlike the test with the ramp of 0.033 K/s.

### 4.2. Free vs. Constrained Sintering

**Figure 5:**
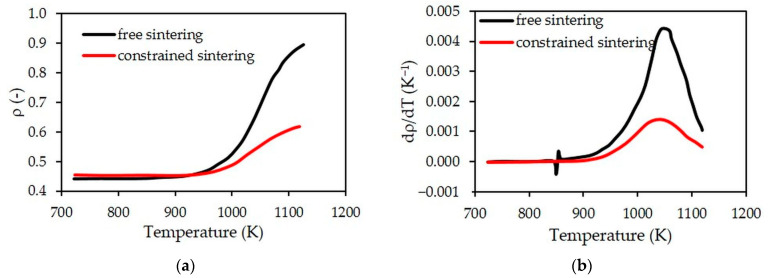
Evolutions in temperature of relative density (**a**) and densification rate (**b**) during free (measured) and constrained (simulated) sintering.

Using the developed numerical model makes it possible to compare densification behaviors during free (experimental values) and constrained sintering (simulated ones). As shown in Figure 5a, the densification process is significantly slowed down during constrained sintering, with a relative density at the beginning of the dwell at 1123 K of 0.63 vs. 0.89 when comparing constrained and free paths. During heating, a discontinuity in the evolution of densification rate for free sintering is noticed around 843 K (Figure 5b). This discontinuity is related to the swelling associated with the transition from alpha to beta quartz at this temperature [25,26]. This phase transition and the associated change in volume are not integrated into the numerical model simulating the constrained sintering of the LTCC bilayer. Moreover, the maximum values of the densification rate are reached at the same temperature (1033 vs. 1050 K for constrained and free sintering, respectively). However, the maximum rate of densification is three times lower in constrained sintering (1.4 × 10^−3^ K^−1^ vs. 4.6 × 10^−3^ K^−1^). This difference in behavior is mainly related to the strong reduction of the in-plane shrinkages (in directions perpendicular to the thickness of the layers) during constrained sintering.

**Figure 6:**
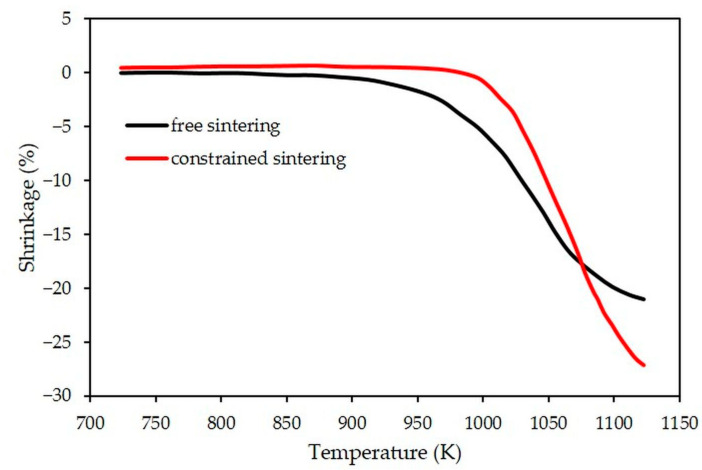
Evolutions in temperature of out-of-plane shrinkage (thickness direction) for free (measured) and constrained (simulated) sintering.

Nevertheless, it should be noticed that shrinkage along the thickness axis is significantly greater after constrained sintering (28% vs. 21%, see Figure 6). This is induced by the generation of tensile stresses in the in-plane directions within the porous LTCC, which leads to an increase of the out-of-plane shrinkage as suggested by the Newtonian viscous law (Equation (11)):

(11) $${\dot{\varepsilon }}_{ij}=\frac{\sigma_{ij}^{\prime }\;}{2G}+\frac{\left(\sigma^{m}-\sigma^{fr}\right)}{3K}\delta_{ij}$$

with *σ′_ij_* the deviation stress, *σ^m^* the volume stress, *σ^fr^* the stress related to the sintering process and *δ_ij_* the Kronecker symbol (if *i* = *j*, *δ_ij_* = 1; if *i* ≠ *j*, *δ_ij_* = 0), *K* the coefficient of viscous compressibility and *G* the deviatory viscosity.

**Figure 7:**
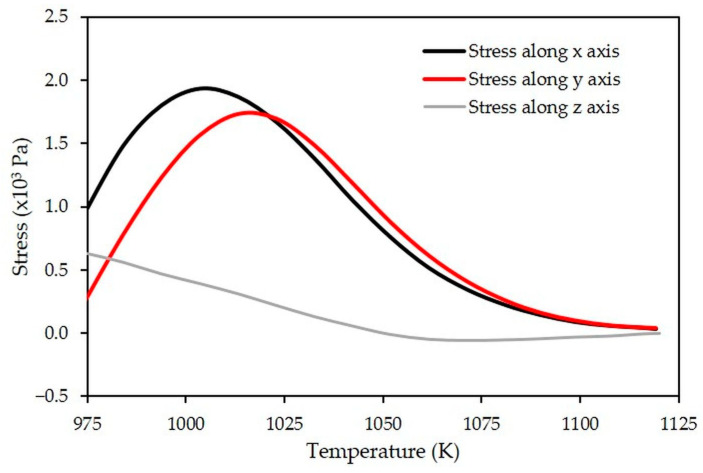
Simulated evolutions of the average stresses induced within the porous LTCC during constrained sintering.

The average stresses occurring within the porous LTCC during constrained sintering are calculated and displayed in Figure 7. At the beginning of the densification of the porous LTCC (above 973 K), the in-plane (x-y) stresses increase similarly up to 1023 K, corresponding to the peak of the densification rate. At 1023 K, they are seven times higher than the out-of-plane stress (along z), and the tensile stresses in the (x-y) plane are approximately 1.7 × 10^3^ Pa. This value must be compared with the hydrostatic stress (*σ_fr_*) of free sintering, which can be estimated by Equation (12) [27]:

(12) $$\sigma_{fr}=\frac{{\dot{\varepsilon }}_{fr}\cdot \eta }{1-2\nu_{p\;}}$$

with *σ_fr_* the hydrostatic stress of free sintering, ${\dot{\varepsilon }}_{fr}$ the rate of strain in free sintering, *η* the viscosity, and *ν_p_* the viscous Poisson’s coefficient.

At 1023 K, the relative density in free sintering is equal to 0.65 (Figure 5a) for a shrinkage rate of approximately −1.9 × 10^−4^ s^−1^ (obtained by plotting the curve shrinkage vs. time, then its derivative). The uniaxial viscosity (*η*) estimated from Equation (9) is then 8.7 × 10^6^ Pa·s. The hydrostatic stress of the LTCC material for free sintering is estimated at 2.3 × 10^3^ Pa when considering the above equation. This result indicates that at 1023 K, the tensile stresses in the (x-y) plane are quite similar to the ones associated with the densification process. So, a strong limitation of the in-plane shrinkages appears. All of these results confirm the hypothesis that the dense LTCC layer leads, in this geometric configuration, to the constrained sintering of the porous LTCC.

**Figure 8:**
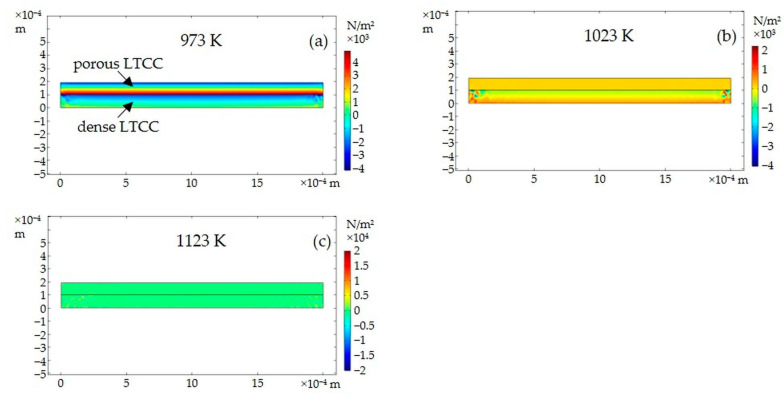
Cross-section distributions of normal stress along the *x*-axis (i.e., direction along the sample length) at mid-length of the LTCC bilayer for different temperatures: (**a**) 973 K, (**b**) 1023 K, and (**c**) 1123 K.

The cross-section distributions of normal stress along the x-axis (i.e., the direction along the sample length) are displayed in Figure 8 for different temperatures. This axis corresponds to the direction of preponderant deformation leading to the curvature of the sample. At the beginning of densification of the porous LTCC (at 973 K, Figure 8a), strong stress gradients are generated within both layers. At the interface between the porous and dense LTCCs, the porous layer sustains tensile stresses while the dense one is in compression. At 1023 K, corresponding approximately to the peak of the densification rate (Figure 8b), stress values are higher in both layers. The porous LTCC undergoes tensile stresses, which are relatively homogeneous over the thickness of the porous LTCC. Conversely, the stress gradient remains within the dense LTCC, going from compressive stresses at the interface with the porous layer to tensile stresses on the free side. At the end of densification (at 1123 K, Figure 8c), the level of stresses within both layers is greatly reduced, being progressively relaxed by creep and apparition of the camber phenomenon.

### 4.3. Evolution of Camber during Constrained Sintering

**Figure 9:**
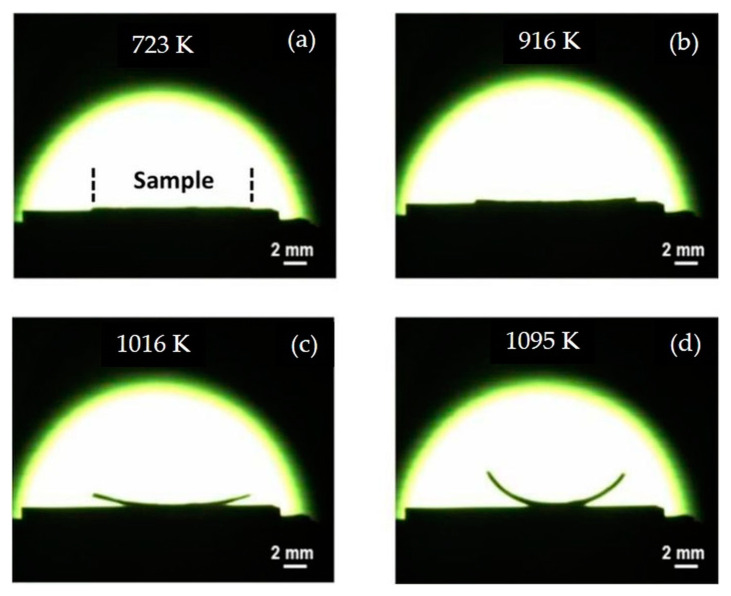
Camber measurements of the bilayer at (**a**) 723 K, (**b**) 916 K, (**c**) 1016 K, and (**d**) 1095 K.

Camber evolution in temperature of the bilayer has been monitored experimentally using the ombroscopy system (Figure 9).

At low temperatures and after calcination at 723 K, no deformation is observed. The bilayer remains perfectly flat (Figure 9a). A low deformation is observed from 916 K (Figure 9b). It is explained by the beginning of densification of the porous LTCC in accordance with the densification curves (Figure 5).

Above 916 K, camber increases monotonously during heating (Figure 9c,d). This distortion is induced by the relaxation of the stresses generated in the joining plane (x-y) of the bilayer during the densification of the porous layer. These stresses are maximum around 1016 K (Figure 7).

The maximum camber is detected at 1095 K, with a deflection at the extreme point of the bilayer equal to 2.75 × 10^−3^ m (Figure 9d).

**Figure 10:**
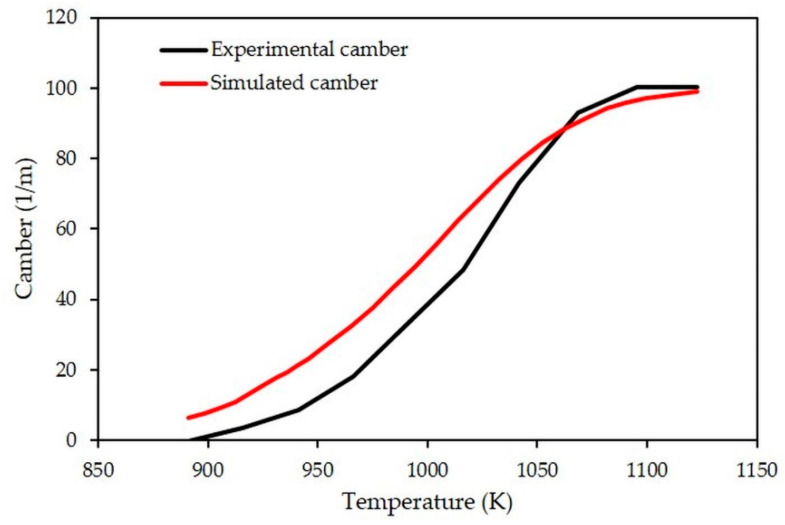
Comparison between simulated and measured evolutions of camber during thermal treatment.

The values of the radius of curvature and the associated camber were determined experimentally from 916 K to 1123 K (Figure 10). At low temperatures, camber is negligible, the radius of curvature tending to infinity. As temperature increases, the camber reaches a maximum value of 100.3 m^−1^ at 1095 K. This camber value can be associated with a curvature radius value of 10.0 × 10^−3^ m.

Numerically, the camber phenomenon is also predicted by the model. The simulated evolution of the camber during heating is compared to that measured experimentally (Figure 10). It appears that distortion starts around the same temperature, close to 916 K. The simulated camber increases exponentially up to about 1033 K before slowing down due to a reduction in the densification rate of the porous LTCC (Figure 5). At 1095 K, the calculated deflection is 2.86 × 10^−3^ m, which is almost identical to that measured by the ombroscopic method (2.75 × 10^−3^ m). The simulated evolution of the camber predicted by the model is therefore close to the measured one, with camber at 1095 K of 97.4 vs. 100.3 m^−1^, respectively. The camber simulated between 898 K and 1048 K is slightly higher than the one measured experimentally. This phenomenon can be explained by a uniaxial viscosity value (Equation (10)) slightly lower than that of the measured values [21].

**Figure 11:**
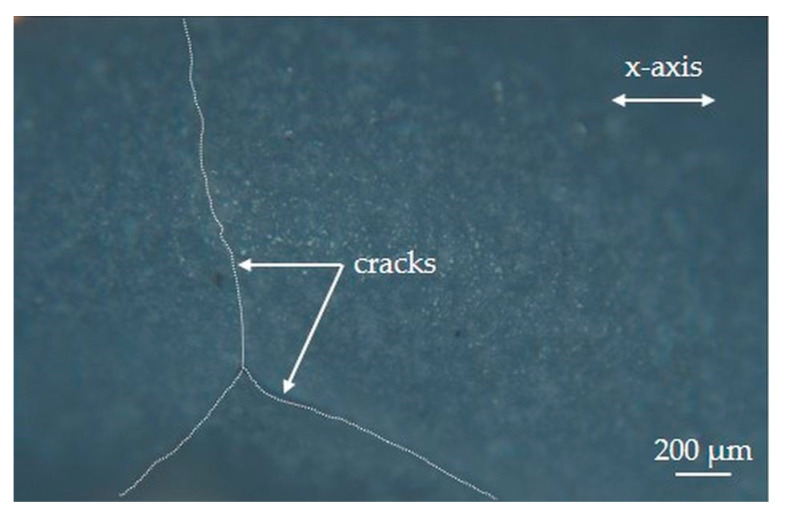
Microcracks (underlined by white dotted lines) observed by optical microscopy on the surface of the dense LTCC layer after heat treatment up to 1123 K. The indicated *x*-axis corresponds to the direction of sample length.

Between 1095 and 1123 K, no significant change in camber is detected experimentally. This stagnation of the camber around 100 m^−1^ over this temperature range seems to be related to the damage of the LTCC ceramic by microcracking at these high temperatures. Indeed, through post-mortem observations by optical microscopy of the surfaces of the LTCC bilayer, some microcracks are visible (Figure 11). They are located rather on the side of the dense layer and at the mid-length of the sample. As the numerical model does not take this mode of damage into account, the simulated camber continues to increase slightly over this temperature range.

## 5. Conclusions

In this work, a numerical model was developed to simulate the constrained sintering of LTCC layers. This finite element model is based on the thermomechanical and sintering behavior laws previously identified for the LTCC material. A methodology combining experiment and simulation is proposed to validate the built model. The distortion generated during the constrained sintering of a porous LTCC layer deposited on a dense one is monitored in situ through ombroscopic imaging. The numerical simulation predicts the evolutions of the camber during thermal treatment of the LTCC-based bilayer with good reliability. It shows the robustness of the developed numerical model. In particular, the applied methodology and associated results validate the use of a linear isotropic viscous constitutive law to describe the viscoplastic behavior of the LTCC material during constrained sintering. The shaping process and the thickness of the LTCC layers (around 100 × 10^−6^ m) do not seem to induce a noticeable anisotropic character in the thermomechanical response of the LTCC material. At high temperatures (above 1095 K), the simulated camber evolution slightly differs from the experimental one due to microcracking of the dense LTCC layer.

## Figures and Tables

**Table 1:** Sintering and viscoplastic parameters of the LTCC material.

Parameters	Settings	Porous LTCC	Dense LTCC
Sintering parameters	*Q_fs_* (J·mol^−1^)	303,000	-
	*ρ*_0_ (-)	0.46	-
	*b* (-) *c* (-)	−15.50 0.48	- -
Viscoplastic parameters	*Q_vp_* (J·mol^−1^)	321,000	114,000
	*η*_0_ (Pa·s) *β* (-) *ν_p_* (-)	1.89 × 10^−9^ 9.23 0.14	2.22 × 10^2^ 0 0.50

## Data Availability

Not applicable.
